# Successful Treatment of a Recurring Rectal Cloacogenic Polyp With Topical Steroids

**DOI:** 10.7759/cureus.57974

**Published:** 2024-04-10

**Authors:** Jeanine Karpf, Fritz R Murray, Peter Bauerfeind

**Affiliations:** 1 Department of Gastroenterology and Hepatology, Stadtspital Zurich, Zurich, CHE; 2 Department of Gastroenterology and Hepatology, Klinik St. Anna, Lucerne, CHE

**Keywords:** mucosal prolapse, bright red blood per rectum, rectal polyps, conventional endoscopic mucosal resection (cmer), cloacogenic polyp

## Abstract

Cloacogenic polyps (CPs) are considered benign lesions arising in the anorectal transition zone. Most, but not all, patients are symptomatic with hematochezia, constipation, or abdominal pain. Although considered benign, resection is recommended due to the possibility of malignant transformation. In the case of recurrent disease, re-resection is usually hampered by scar tissue. We present the case of a 15-year-old male patient with a refractory CP, eventually successfully treated with topical steroids.

## Introduction

Cloacogenic polyps (CPs) are considered benign lesions arising in the anorectal transition zone [1]. The pathophysiology remains unclear. However, a similar genesis to other conditions belonging to the mucosal prolapse syndrome, such as solitary rectal ulcer syndrome, is assumed [1-3]. Hence, excessive straining with repetitive mucosal prolapse during defecation might play a role. Endoscopically, CP may occur as solitary or multiple and may appear sessile or pedunculated with sizes up to 5 cm and a potentially irregular pit pattern, mimicking anorectal malignancy [2,4-6]. CPs occur more often in adults between 30 and 60 years of age, with male dominance, but have been described in children [2,4]. Most patients are symptomatic, with hematochezia, constipation, or abdominal pain, however, 20% are asymptomatic [2,4].

The natural course of CP is not completely understood. Due to the possibility of malignant transformation, resection followed by endoscopic surveillance for potential recurrence is recommended [7,8].

We hereby present a case of a teenager with daily mucus discharge diagnosed with a recurring CP, eventually successfully treated with topical steroids.

## Case presentation

A 15-year-old, otherwise healthy, male patient presented with daily stool irregularities without concomitant abdominal pain. He reported five to nine bowel movements per day with rather normal consistency. However, each bowel movement was accompanied with anal mucus discharge.

**Figure 1:**
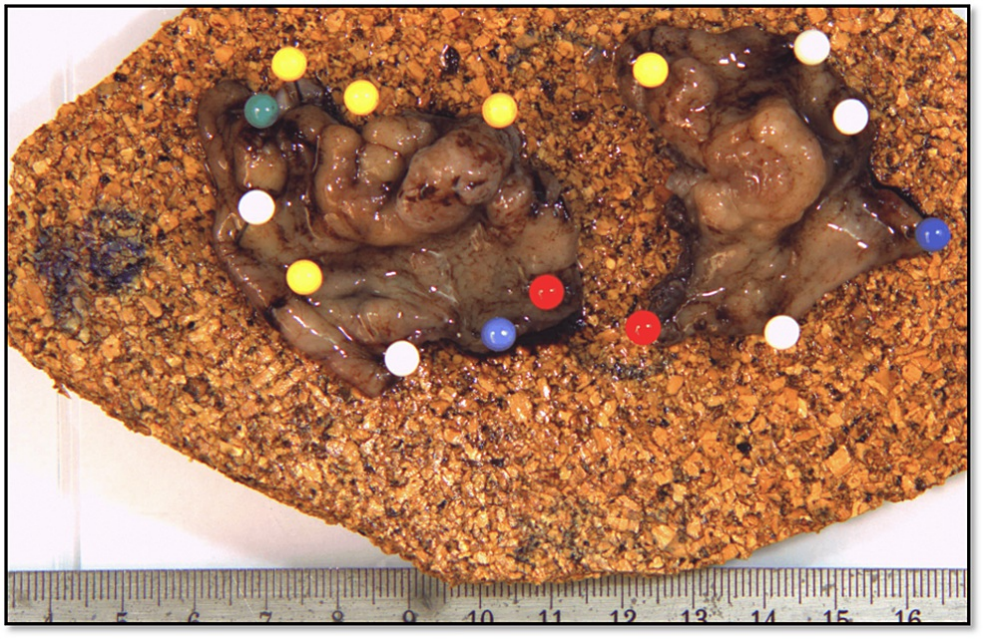
Surgical specimen

Inspection revealed no anal abnormalities. However, a polypoid lesion was palpated in the digital rectal examination. Endoscopically, this was identified as a sessile polyp occupying about three-quarters of the circumference, located about 3 cm proximal to the dentate line. Therapeutically, an uneventful, surgical, transanal, full thickness resection was conducted. The histological examination of the 4.3x3.3x0.5 cm, large resection specimen (Figure 1) characterized the lesion as an inflammatory cloacogenic polyp without signs of dysplasia or malignancy.

**Figure 2:**
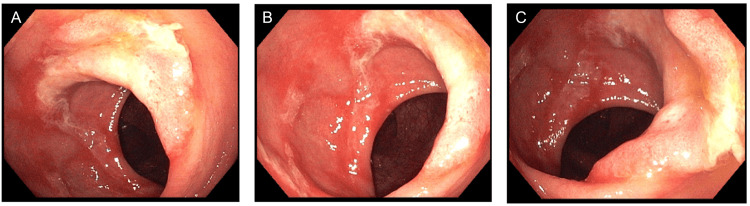
Recurrent polyp three years after surgical resection A-C represent images of the recurring polyp from different angles during rectosigmoidoscopy

Three years after the initial resection, the patient presented again with daily anal mucus discharge. Endoscopically, a recurrent sessile polyp 1 cm proximal to the dentate line was identified (Figure 2). Biopsies were positive for the recurrence of an inflammatory polyp.

Endoscopic resection was discussed but denied by the patient. Instead, a once-daily topical steroid therapy with a Procto-Synalar® suppository, consisting of Fluocinolone acetonide (0.1 mg) and Lidocaine hydrochloride-1-water (40 mg), was started.

**Figure 3:**
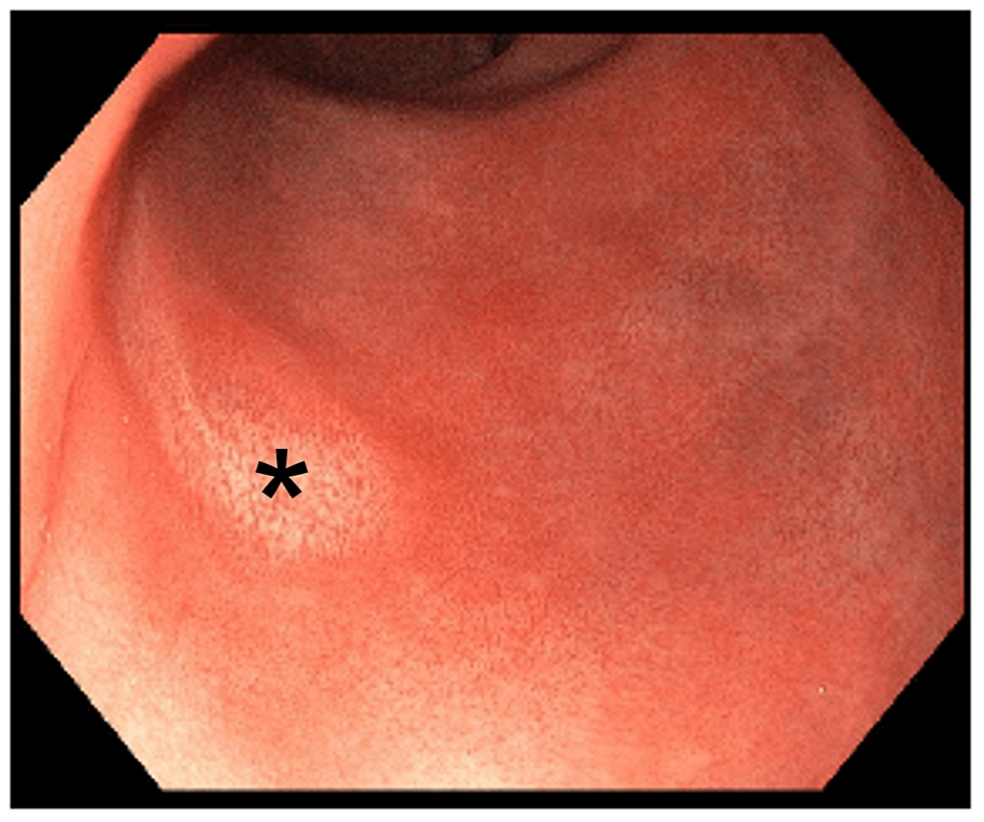
Endoscopic image three months after topical steroid therapy The former site of the polyp (indicated by an asterisk) macroscopically appears somewhat lighter than the surrounding mucosa. The area was biopsied without the histological features of a residual polyp.

**Figure 4:**
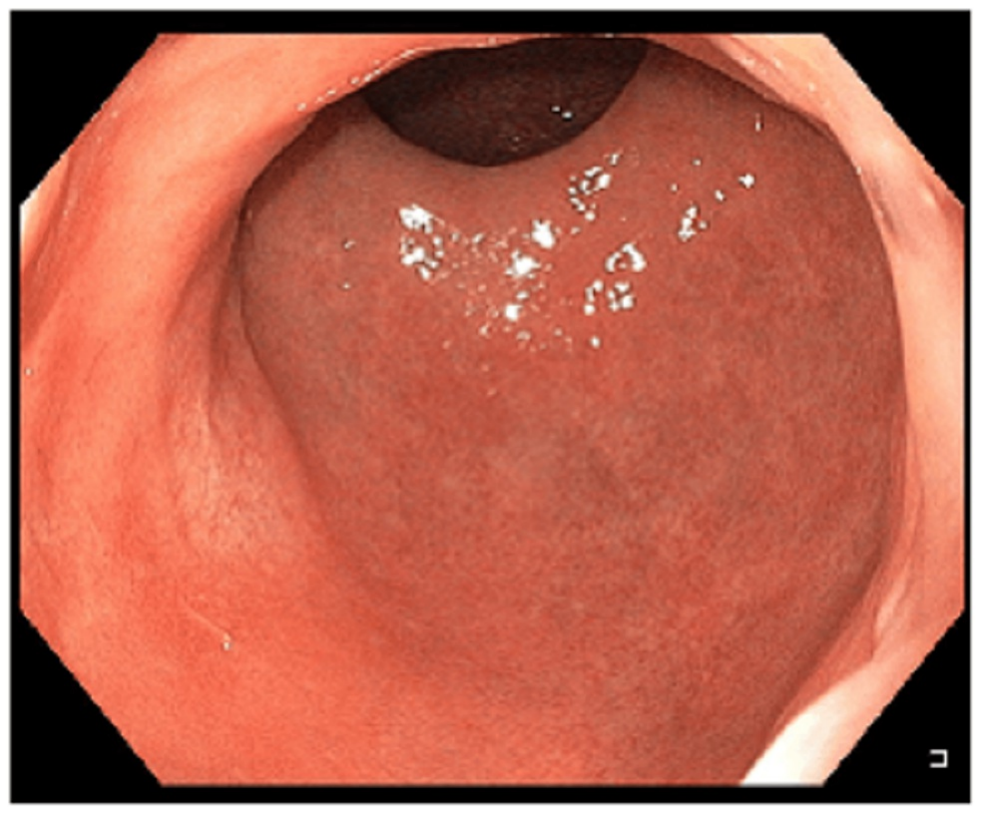
Endoscopic image six months after topical steroid therapy

After three months of consistent therapy, the fully asymptomatic patient was seen for an endoscopic follow-up. A rectosigmoidoscopy was conducted in which no residual polyp could be identified (Figure 3). A biopsy of the former site of the polyp, which macroscopically appeared somewhat lighter than the surrounding mucosa, was negative. Six months (Figure 4) and again two years after the end of topical steroid therapy, no recurrence could be detected endoscopically, so that complete healing could be assumed.

## Discussion

In this case report, we demonstrated a successful treatment with a complete resolution of a cloacogenic polyp in the rectum with topical steroids.

While most often symptomatic, even in asymptomatic cases, treatment is suggested due to potential malignant transformation. As a general measure, constipation and straining ought to be treated with a high-fiber diet, laxative treatment, and/or biofeedback for pelvic floor disorders to prevent recurrence. Nowadays, resection is usually conducted endoscopically [4] while size, location, and the number of polyps as well as the individual endoscope skill set of the operator determines the resection technique. Most often, conventional cold snare polypectomy is conducted due to its broad availability, cost-effectiveness, and low complication risk [4,9]. Just as in rectal adenomas and even early rectal cancer, endoscopic submucosal dissection (ESD) has been successfully attempted [5,10]. Depending on the polyp geometry and size, ESD has the advantage of higher en-bloc resection rates and minimizes the risk for recurrence [11]. Especially in cases when rectal polyps are large and/or have macroscopic features of deep mucosal invasion, ESD is becoming more popular [12]. However, ESD is only available in expert centers, has a shallow learning curve, is time-consuming, and is associated with slightly higher rates of adverse events [11]. In addition, ESD is generally considered technically more complicated or even not possible in recurrent disease following an initial resection or even biopsy due to the formation of scar tissue [13]. Bearing in mind that CP may macroscopically have high-risk features or may even mimic malignant disease, endoscopic resection during index endoscopy seems natural. However, in the case of recurrent disease, no guidelines regarding further treatment exist. As mentioned above, re-resection is usually hampered by scar tissue. Alternatively, an endoscopic full thickness or even surgical resection is possible. However, especially later entails the risk of injury to the anorectal sphincter.

Topical steroids are regularly used in the treatment of inflammatory conditions in the gastrointestinal tract as well as in solitary rectal ulcer syndrome. The mode of action in CP may be explained by the histological features, consisting (among others) of epithelial reparative changes, continuous destructive regenerative processes, and mixed inflammatory cell infiltration [2].

## Conclusions

To the best of our knowledge, successful steroid treatment of a CP has not been described before. It represents a valuable, safe, and cheap option, especially in recurrent disease or whenever patients are not willing to undergo (repeated) resection or are not fit for surgery.
